# Mutant Nrf2^E79Q^ enhances the promotion and progression of a subset of oncogenic Ras keratinocytes and skin tumors

**DOI:** 10.1016/j.redox.2024.103261

**Published:** 2024-06-28

**Authors:** John G. Witherspoon, Jonathan R. Hall, Dereje Jima, Hannah M. Atkins, Nathan T. Wamsley, Michael B. Major, Bernard E. Weissman, Robert C. Smart

**Affiliations:** a Department of Biological Sciences, North Carolina State University, USA; b Toxicology Graduate Program, North Carolina State University, USA; c Center for Human Health and the Environment, North Carolina State University, USA; d Department of Cell Biology and Physiology, Washington University at St Louis, USA; e Department of Pathology and Laboratory Medicine, University of North Carolina at Chapel Hill School of Medicine, USA; f Lineberger Comprehensive Cancer Center, University of North Carolina at Chapel Hill School of Medicine, USA

**Keywords:** Nrf2, NFE2L2, KEAP1, Mouse skin multistage carcinogenesis model, Ras, Papilloma, Squamous cell carcinoma, Proteomics, RNAseq, Cancer, Progression, Conversion, Promotion

## Abstract

Squamous cell carcinomas (SCCs), including lung, head & neck, bladder, and skin SCCs often display constitutive activation of the KEAP1-NRF2 pathway. Constitutive activation is achieved through multiple mechanisms, including activating mutations in *NFE2L2* (NRF2). To determine the functional consequences of Nrf2 activation on skin SCC development, we assessed the effects of mutant Nrf2^E79Q^ expression, one of the most common activating mutations in human SCCs, on tumor promotion and progression in the mouse skin multistage carcinogenesis model using a DMBA-initiation/TPA-promotion protocol where the *Hras* A->T mutation (Q61L) is the canonical driver mutation. Nrf2^E79Q^ expression was temporally and conditionally activated in the epidermis at two stages of tumor development: 1) after DMBA initiation in the epidermis but before cutaneous tumor development and 2) in pre-existing DMBA-initiated/TPA-promoted squamous papillomas. Expression of Nrf2^E79Q^ in the epidermis after DMBA initiation but before tumor occurrence inhibited the development/promotion of 70% of squamous papillomas. However, the remaining papillomas often displayed non-canonical *Hras* and *Kras* mutations and enhanced progression to SCCs compared to control mice expressing wildtype Nrf2. Nrf2^E79Q^ expression in pre-existing tumors caused rapid regression of 60% of papillomas. The remaining papillomas displayed the expected canonical *Hras* A->T mutation (Q61L) and enhanced progression to SCCs. These results demonstrate that mutant Nrf2^E79Q^ enhances the promotion and progression of a subset of skin tumors and alters the frequency and diversity of oncogenic Ras mutations when expressed early after initiation.

## Graphical abstract

**Figure:**
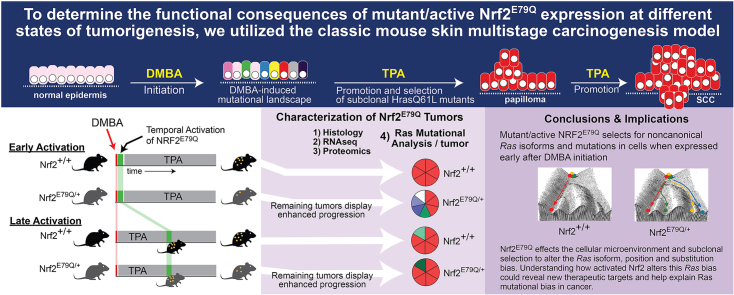

## 1 Introduction

The *NFE2L2* gene (hereafter *NRF2*) encodes a transcription factor, NRF2, that, when active, regulates a cytoprotective gene expression program to control redox biology, oxidative stress, xenobiotic metabolism and excretion, iron and amino acid metabolism, and mitochondrial bioenergetics [[1], [2], [3]]. In the absence of stress, NRF2 protein is kept at low levels through KEAP1/CUL3-dependent ubiquitylation and subsequent NRF2 proteasomal degradation [4]. Cellular stress, such as increased xenobiotic electrophiles or reactive oxygen species (ROS) results in the chemical modification of cysteines within KEAP1, stabilizing NRF2 and allowing translocation of NRF2 to the nucleus where it activates the transcription of NRF2 target genes harboring ARE/EpRE elements (antioxidant response element/electrophile-responsive element) [5,6].

Many studies have shown a protective role for NRF2 in chemical and radiation-induced tumorigenesis using genetically engineered mouse models (GEMM) or treatment with chemopreventive agents that stabilize NRF2 protein [[7], [8], [9], [10]]. However, squamous cell carcinomas (SCCs) from a variety of human epithelial tissues, including lung, esophagus, head and neck, and skin, often display constitutive activation of the KEAP1-NRF2 pathway through numerous mechanisms, including *NRF2* activating mutations (e.g., NRF2^E79Q^), amplification of the *NRF2* genomic locus and loss of function mutations or deletions in the *KEAP1* or *CUL3* genes [2,[11], [12], [13], [14], [15], [16], [17], [18], [19]]. Patients with lung squamous cell carcinoma (SCC) carrying mutant/active NRF2 have a poor prognosis compared to patients with lung SCC with wildtype NRF2 [20]. It is generally accepted that tumor cells must overcome the cellular stresses associated with tumorigenesis to acquire the hallmark traits of cancer [21,22]. These cellular stresses, including oxidative, mitotic, proteotoxic, and metabolic are referred to as the “stress phenotypes of tumorigenesis/cancer” [22]. The activation of NRF2 pathways during tumorigenesis is generally thought to promote an adaptability and provide an evolutionary advantage to overcome the many stressors inherent to oncogenesis. However, the impact of the timing of the mutational activation of the KEAP1-NRF2 pathway and its consequences on tumor development/promotion and progression are poorly understood. Recent studies have also shown that the implicated dose of NRF2 activity also impacts tumor initiation and promotion [23].

To investigate the function consequences of the hotspot mutant/active Nrf2^E79Q^ at different stages of tumorigenesis, we utilized the classic mouse skin multistage carcinogenesis model [24,25]. In this model, mouse skin is initiated with a single topical treatment of the mutagenic polycyclic aromatic hydrocarbon, 7,12-dimethylbenz[*a*]anthracene (DMBA), leading to DMBA's canonical *Hras* A->T (Q61L) mutation [26,27]. The *Hras* A->T (Q61L) mutation is the consequence of DMBA treatment as it is detected in epidermal keratinocytes shortly after DMBA treatment [28,29]. The DMBA-initiated skin remains morphologically normal until weekly topical treatments with the tumor promoter 12-*O*-tetradecanoylphorbol-13-acetate (TPA) [25] which promotes the development of squamous papillomas. Almost all (>95%) of the resulting squamous papillomas contain the canonical *Hras* A->T (Q61L) driver mutation [[25], [26], [27],^30^]. Some oncogenic *Hras* squamous papillomas subsequently progress to SSCs [25]. While DMBA significantly alters the mutational landscape in the epidermis [[31], [32], [33]], a selection for the clonal expansion of keratinocytes with canonical A->T transversion in *Hras* (Q61L) occurs in the DMBA-initiated/TPA-promoted mouse model.

Here, we used our K14CreER^tam^*;*LSL-Nrf2^E79Q/wt^ GEMM(34), which conditionally expresses one of the most common *NRF2* mutations found in human tumors. Using K14CreER^tam^;LSL-Nrf2^E79Q/wt^ mice to allow for temporal control over *Nrf2*^*E79Q*^ expression, we examined the effect of NRF2^E79Q^ on: 1) skin tumor development after DMBA initiation in the epidermis but before cutaneous tumor development, and 2) in pre-existing DMBA-initiated/TPA promoted squamous papillomas. Our results indicate mutant NRF2^E79Q^ enhances the promotion and progression of a subset of oncogenic Ras keratinocytes and skin tumors. When expressed early after initiation, mutant NRF2 alters the Ras isoform, position and substitution bias in tumors.

## 2 Methods

### 2.1 Mice and tumor experiment

The knockin LSL-Nrf2^E79Q/wt^ mouse has the mutant *Nrf2*^*E79Q*^ allele knocked into the endogenous *Nrf2* locus, where in the absence of Cre activation it remains silenced by an LSL Stop cassette [34]. The K14CreER^tam^ mouse directs CreER^tam^ transgene expression to the epidermis via the K14 promoter, and upon tamoxifen (TMX) treatment, CreER^tam^ recombinase is activated [35]. The LSL-Nrf2^E79Q/wt^ C57BL/6J;129S1/SvImJ hybrid mice and K14CreER^tam^ C57BL/6J;129S1/SvImJ hybrid mice [35] were crossed to generate LSL-Nrf2^E79Q/wt^, K14CreER^tam^, and K14CreER^tam^;LSL-Nrf2^E79Q/wt^ C57BL/6J;129S1/SvImJ hybrid mice. Male and female mice aged 8–12 weeks were randomly assigned to the treatment groups, with a similar number of male and female mice in each treatment group. The mice had their dorsal hair clipped with electric clippers and were given one topical dose of 200 nmol 7,12-dimethylbenz[*a*]anthracene (DMBA) (Thermo Fisher Scientific Cat. No. 408181000) (0.2 ml) in acetone.

For tumor studies examining the effect of mutant Nrf2^E79Q^ expression early on tumor development after DMBA treatment, mice received 2.5 mg TMX (Sigma-Aldrich Cat. No. T5648), dissolved in corn oil with 5% ethanol daily (0.25 ml) intraperitoneally (i.p.) 5 days/week for 2 weeks beginning one week after DMBA initiation, after which the mice were treated topically thrice weekly with 10 nmol 12-*O*-tetradecanoylphorbol-13-acetate (TPA) (LC Laboratories Cat. No. P-1680) (0.2 ml) in acetone for 40 weeks. For the tumor study in which mutant Nrf2 expression was activated in pre-existing tumors, the mice one week after DMBA treatment were treated topically thrice weekly with 10 nmol TPA (0.2 ml) in acetone for 40 weeks. At 20 weeks of TPA treatment, all mice were treated (i.p.) with 2.5 mg TMX dissolved in corn oil with 5% ethanol daily (0.25 ml), 5 days/week for 2 weeks while maintaining the TPA treatments. All palpable tumors (exophytic and sessile) on the dorsal skin were counted every two weeks, and multiplicity and incidence were determined. Tumor with diameters ≥1 mm were measured at the end of the study. All aspects of animal care and experimentation described in this study were conducted according to the NIH guidelines and were approved by the NC State University Institutional Animal Care and Use Committee. NC State University is an AAALAC International accredited institution.

### 2.2 Genotyping

Genomic DNA was extracted from mouse tail using GeneJET Genomic DNA Purification Kit (Thermo Fisher Scientific Cat. No. KO721). Mice were genotyped using Taq Polymerase (QIAGEN Cat. No, 201203) for LSL-Nrf2^E79Q^ with primers Nfe2l2 ScF3 (5′-GAT GCC TTC TTC TTG CCT GTA G-3′), Nfe2l2 ScR3 (TCC ACA CGG GTT AGT TCA CTA CA-3′), and AdSA-R (5′-AAA GGG ACA GGA TAA GTA TGA CAT CAT C-3′) [34]. Mice were genotyped for K14CreER^tam^ using Taq Polymerase with primers Cre-1 (5′- CGA TGC AAC GAG TGA TGA GGT TC -3′) and Cre-2 (5′- GCA CGT TCA CCG GCA TCA AC-3′).

### 2.3 Tumor histopathology

Whole dorsal skin with tumors was harvested from all mice at necropsy, laid flat on card stock, fixed in 10% phosphate-buffered formalin and then transferred to 70% ethanol after 24 hours. Macroscopically visible tumors (≥1 mm) and full-thickness adjacent skin were collected from the dorsal skin, maintaining a consistent cranial to caudal longitudinal orientation in the direction of hair growth. Tissues were embedded with paraffin using standard processing settings and then sectioned at 5 μm onto charged slides. Slides were stained with routine hematoxylin and eosin (H&E) stains using an XL Autostainer (Leica Biosystems). Visible tumors larger than 1 mm were diagnosed by a veterinary pathologist (HMA) using standard INHAND diagnostic criteria [36]. In addition, papillomas with squamous cell carcinomas (pap w/SCC) were defined as a papilloma with a region or regions characteristic of a typical squamous cell carcinoma in which neoplastic cells invade past the natural epidermal basement membrane and exhibit other features of malignancy, including increased numbers of often bizarre mitotic figures with nuclear atypia, variable keratinization, and differentiation.

### 2.4 RNA collection

Total RNA was extracted from whole tumors collected from mice one week following the cessation of 40 weeks of TPA treatment. Tumors were homogenized in QIAzol Lysis Reagent (QIAGEN Cat. No. 79306) and RNA was purified using a Zymo Scientific silica-based spin column (Zymo Research Cat. Nom. R1018) and treated with DNase 1 (Zymo Research Cat. No. E1010). K14CreER^tam^ and K14CreER^tam^;LSL-Nrf2^E79Q/wt^ mice were treated with 2.5 mg tamoxifen i.p. (1x/day for 5 days/week for 2 weeks) and then treated topically with DMSO or 50ug CDDO-Methyl/200 μl DMSO (bardoxolone methyl) (Selleck Chemicals Cat No. S8078) 1x/day for 3 days. Twenty-four hours later, the epidermis was collected from whole skin at necropsy via heat shock [30], and epidermal RNA was extracted as described above for tumors.

### 2.5 RNAseq

Illumina RNA library construction and sequencing (20 M 150bp paired-end reads/sample) of epidermal and tumor RNA was conducted by Novogene. Data analysis was performed in consultation with the Bioinformatics Core at NC State's Center for Human Health and the Environment. An average of ∼28.5 million paired-end raw RNAseq data were generated for each replicate. The quality of sequenced data was assessed using the fastqc application, and 12 poor-quality bases were trimmed from the 5′-end. The remaining good-quality reads were aligned to the Mouse reference genome (mm39) downloaded from the Ensembl database using STAR aligner [37]. Per-gene counts of uniquely mapped reads for each replicate were calculated using the htseq-count script from the HTSeq Python package. The count matrix was imported to the R statistical computing environment for further analysis. Initially, genes that had no count in most replicate samples were discarded. The remaining count data were normalized for sequencing depth and distortion, and dispersion was estimated using DESeq2 Bioconductor package in the R statistical computing environment [38]. We fitted a leaner model using the treatment levels, and differentially expressed genes were identified after applying multiple testing corrections using the Benjamini-Hochberg procedure [39]. The final significant genes were generated using an adjusted p-value≤0.05. RNAseq data from early and late expression of mutant NRF2 were analyzed through the use of ingenuity pathway analysis (QIAGEN) to identify canonical pathways, upstream regulators and associated functions related to the expression of mutant NRF2 in mouse epidermis and DMBA/TPA induced skin tumors. Data was analyzed using Right tailed Fisher Exact Test with Benjamini-Hochberg (B–H) multiple hypothesis testing-corrected p-value. Data were filtered by BH adjusted p-value≤0.05 and an absolute z-score of 2.

### 2.6 Detection of *Ras* and *Nrf2* mutations

Mutant allele expression was determined via Integrative Genomics Viewer [40,41] analysis of the .bam files from the aligned RNAseq reads, documenting all differences of the alignments to mm39 with a coverage allele-fraction threshold of 0.15 (≥15% of aligned bases differ) in the case of driver mutations of interest and with no threshold for *Nrf2*^*E79Q*^ (all detectable transgene transcripts reported), excluding single nucleotide polymorphisms.

### 2.7 Targeted mass spectrometry analysis

Proteins were extracted from fresh frozen tissues as described in Wamsley and colleagues [42]. Briefly, protein was extracted on ice by vortexing and manual grinding with a micro pestle in an aqueous solution of 8 M urea, 75 mM NaCl, 50 mM Tris (pH 8.0) and 1 mM EDTA with addition of phosphatase and protease inhibitor cocktails (Halt, catalog no. 78429; 78420). Samples were then digested using Lysyl endopeptidase (Wako Chemicals, 12902541) and trypsin (Promega, PR-V5113). Peptides were desalted by SDB-RPS spin columns (Affinisep, Spin-RPS-M.T1.96) and quantified by a bicinchoninic acid protein assay (Thermo Fisher Scientific, catalog no. 23225).

A total of 1 μg of endogenous tryptic peptides per run were separated by reverse-phase nano-high performance liquid chromatography (HPLC) and analyzed using an Orbitrap Eclipse Tribrid mass spectrometer (Thermo Fisher Scientific). A custom Optimized-Internal-Standard Parallel Reaction Monitoring targeted mass spectrometry (OIS-PRM) method was used as reported previously [42]. Stable isotope labeled (SIL) internal standard peptides are cataloged in Supplementary Table S1 and were injected at a nominal abundance 150 fmol each for every 1 μg of endogenous peptide. Peak area ratios and chromatogram plots for internal standard triggered parallel reaction monitoring (IS-PRM) data were generated using an in-house tool as described [42], but without normalization. To obtain NRF2 scores for each tumor, a PCA analysis was performed on the expression of NRF2 targets, 6PGD, AL3A1, BLVRB, ENTP1, G6PD1, GSH1, HTAI1, HYEP, NQOT, XCT, and NFE2L2. The position of each tumor along the first principal component was reported as the NRF2 score.

## 3 Results

### 3.1 Activation of mutant Nrf2^E79Q^ expression in mouse epidermis specifically activates NRF2 signaling

**Fig. 1:**
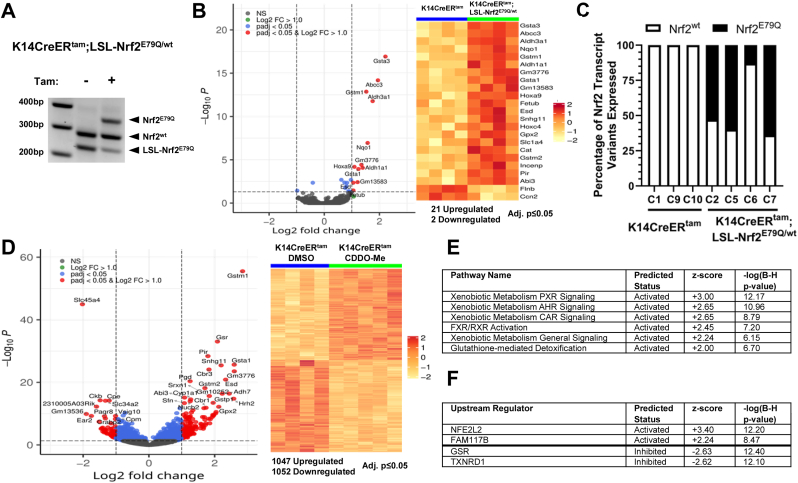
The K14CreER^tam^;LSL-Nrf2^E79Q/wt^ mouse is a tractable system to study *Nrf2*^*E79Q*^ activation in tumor development and progression. **(A)** TMX-treated K14CreER^tam^;LSL-Nrf2^E79Q/wt^ mice display Cre-mediated recombination of *LSL-Nrf2*^*E79Q*^. **(B)** Volcano Plot and heatmap of epidermal RNAseq data show altered gene expression in TMX-treated K14CreER^tam^;LSL-Nrf2^E79Q/wt^ mice when compared to the TMX-treated K14CreER^tam^ mice. Each column represents epidermis from a single mouse. **(C)** Tamoxifen treated K14CreER^tam^;LSL-Nrf2^E79Q/wt^ epidermis expresses *Nrf2*^*E79Q*^ transcripts. Each bar represents a single mouse. **(D)** Volcano plot and heatmap of epidermal RNAseq data show altered gene expression in CDDO-Me treated K14CreER^tam^ mice compared to vehicle (DMSO) K14CreER^tam^ mice. Each column represents a single mouse. **(E)** IPA pathway analysis of RNAseq data reveals, of the significantly enriched pathways, the top pathways predicted to be activated (z-score ≥2.0 are predicted to be activated) in epidermis of TMX-treated K14CreER^tam^;LSL-Nrf2^E79Q/wt^ mice compared to TMX-treated K14CreER^tam^ mice. Right tailed Fisher Exact Test with Benjamini-Hochberg (B–H) multiple hypothesis testing-corrected p-value **(F)** IPA's upstream regulator analysis of RNAseq data reveals top upstream transcription regulators, upstream regulators with z-scores ≥2.0 are predicted to be activated and upstream regulators with z-scores ≤ −2.0 are predicted to be inhibited based on observed gene expression changes in epidermis of TMX-treated K14CreER^tam^;LSL-Nrf2^E79Q/wt^ mice compared to TMX-treated K14CreER^tam^ mice. Right tailed Fisher Exact Test with Benjamini-Hochberg (B–H) multiple hypothesis testing-corrected p-value.

We developed an LSL knockin K14CreER^tam^;LSL-Nrf2^E79Q/wt^ mouse model to investigate the functional consequences of the temporal activation of mutant *Nrf2*^*E79Q*^ expression at different stages of skin tumor development using the mouse skin multistage carcinogenesis model. As shown in Fig. 1A, dosing K14CreER^tam^;LSL-Nrf2^E79Q/wt^ mice with 2.5 mg tamoxifen(TMX) i.p. (1x/day for 5 days/week for 2 weeks) resulted in recombination of the LSL-Nrf2^E79Q^ allele to remove the LSL cassette and produce NRF2^E79Q^ in the epidermis. The residual unrecombined LSL-Nrf2 allele is likely due to the contribution of non-keratinocyte non-K14 expressing cell types. No recombination was observed in the epidermis of untreated K14CreER^tam^;LSL-Nrf2^E79Q/wt^ mice. TMX-treated K14CreER^tam^;LSL-Nrf2^E79Q/wt^ mice demonstrated a 2–3 fold increase in *Nqo1* transcripts (data not shown), an NRF2 target gene, in their epidermis compared to untreated K14CreER^tam^;LSL-Nrf2^E79Q/wt^ mice confirming the activation of NRF2 signaling pathway.

To determine the effect of mutant NRF2 on the epidermal transcriptome, we conducted RNAseq on the epidermis of TMX-treated K14CreER^tam^;LSL-Nrf2^E79Q/wt^, and TMX-treated K14CreER^tam^ mice. We also compared K14CreER^tam^ mice treated with TMX with K14CreER^tam^ mice treated with CDDO-Me as a positive control. CDDO-Me is a potent inhibitor of KEAP1, leading to potent activation of the NRF2 pathway. We identified 23 genes (21 upregulated and 2 downregulated) out of a data set of 14,102 genes that were altered in TMX-treated K14CreER^tam^;LSL-Nrf2^E79Q/wt^ epidermis compared with the TMX-treated K14CreER^tam^ mouse epidermis (adj. p ≤ 0.05) (Fig. 1B, Supplementary Table 1). Integrative Genomics Viewer (IGV) analysis of *Nrf2* transcripts confirmed expression of mutant and wild type *Nrf2* transcripts in the epidermis of the TMX-treated K14CreER^tam^;LSL-Nrf2^E79Q/wt^ mice (average of 47% of mutant *Nrf2* transcripts across the four TMX-treated K14CreER^tam^;LSL-Nrf2^E79Q/wt^ mice); only wildtype *Nrf2* transcripts were found in the K14CreER^tam^ group (Fig. 1C). In contrast to the limited number of changes in gene expression after Nrf2^E79Q^ activation, we identified 2099 genes (1047 upregulated and 1052 downregulated) that were significantly changed in the epidermis by CDDO-Me treatment (adj. p ≤ 0.05) (Fig. 1D–Supplementary Table 2). The significantly changed genes in the CDDO-Me treated mice contained all but one (Hoxc4) of 23 genes that were significantly changed in TMX-treated K14CreER^tam^;LSL-Nrf2^E79Q/wt^ mice.

Ingenuity Pathway Analysis (IPA) of the RNAseq data set (adj. p ≤ 0.05) from the epidermis of TMX-treated K14CreER^tam^;LSL-Nrf2^E79Q/wt^ compared to the epidermis of TMX-treated K14CreER^tam^ mice revealed the most enriched canonical pathway was xenobiotic metabolism signaling, and of the significantly enriched pathways, the top pathways predicted to be activated (z-score ≥2.0) were all pathways associated with NRF2 activation, these include xenobiotic metabolism PXR signaling pathway, xenobiotic metabolism AHR signaling pathway and glutathione-mediated detoxification (Fig. 1E–Supplementary Table 3). IPA's Upstream Regulator Analysis identified NFE2L2 (NRF2) as the top upstream transcription regulator with the highest activation z-score that explained our dataset's observed gene expression changes (Fig. 1F–Supplementary Table 4). These results indicate that the K14CreER^tam^;LSL-Nrf2^E79Q/wt^ mouse is a tractable model to test the functional consequences of activating NRF2 during different stages of tumor development.

### 3.2 Activation of mutant Nrf2^E79Q^ expression after DMBA initiation inhibits skin tumor promotion/development

**Fig. 2:**
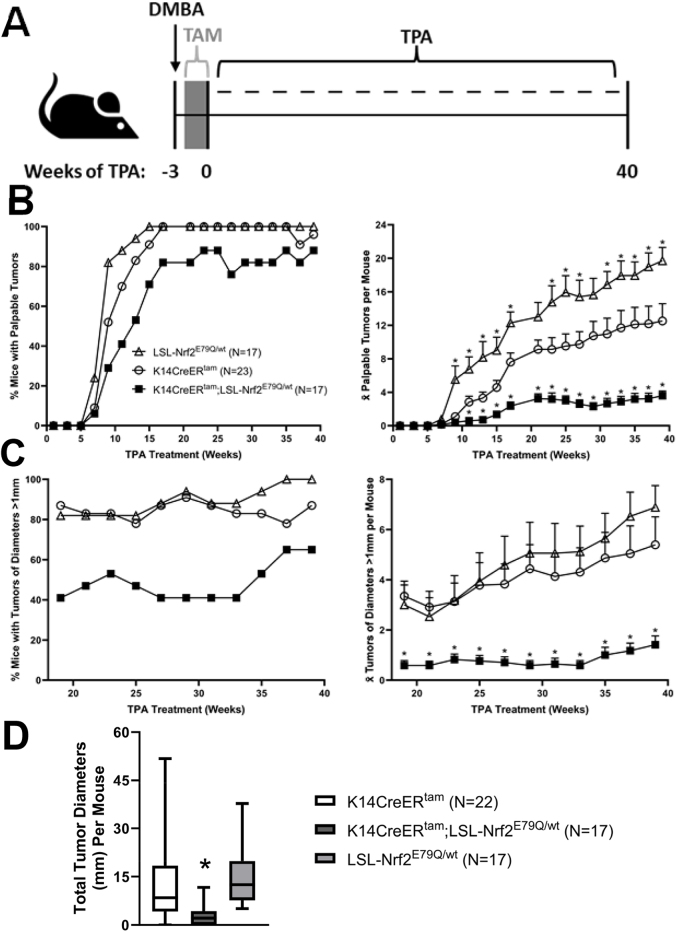
Activation of *Nrf2*^*E79Q*^ expression after DMBA treatment but before tumor development decreases tumor development. **(A)** K14CreER^tam^, K14CreER^tam^;LSL-Nrf2^E79Q/wt^ and LSL-Nrf2^E79Q/wt^ mice were initiated with a single topical application of 200 nmol DMBA. One week later the mice were dosed with 2.5 mg TMX i.p. once a day, 5 days a week for two weeks. One week after the cessation of TMX treatment the mice were promoted with 10 nmol TPA thrice weekly for 40 weeks. Tumors were measured and counted every two weeks during TPA promotion. After 40 weeks of promotion tumors and whole epidermis were collected for histological, protein, and RNA analysis. **(B)** The incidence and average number of all palpable skin tumors per mouse according to genotype. K14CreER^tam^;LSL-Nrf2^E79Q/wt^ mice had significantly less tumors than K14CreER^tam^ (p ≤ 0.05). LSL-Nrf2^E79Q/wt^ mice had significantly more tumors than K14CreER^tam^ (p ≤ 0.05). * Denotes p ≤ 0.05 Student's t-test. **(C)** The incidence and average number of skin tumors >1 mm^3^ per mouse according to genotype. K14CreER^tam^;LSL-Nrf2^E79Q/wt^ mice had significantly less tumors than K14CreER^tam^ (p ≤ 0.05). LSL-Nrf2^E79Q/wt^ mice did not have significantly more tumors than K14CreER^tam^. *Denotes p ≤ 0.05 Student's t-test. **(D)** Diameters of skin tumors ≥1 mm per mouse were measured at 39 weeks and grouped according to genotype. The tumor diameters of K14CreER^tam^;LSL-Nrf2^E79Q/wt^ mice were significantly less than K14CreER^tam^ and LSL-Nrf2^E79Q/wt^ mice. *Denotes p ≤ 0.05 Student's t-test.

To determine the effect of mutant NRF2^E79Q^ on tumor promotion, K14CreER^tam^, K14CreER^tam^;LSL-Nrf2^E79Q/wt^ and LSL-Nrf2^E79Q/wt^ mice were treated as shown in Fig. 2A. Briefly, all mice were initiated with DMBA, one week later, mice were treated with TMX to remove LSL cassette, and one week after cessation of TMX, mice were treated with TPA 3x/week for 40 weeks. After 17 weeks of TPA treatment, 100% of K14CreER^tam^ and LSL-Nrf2^E79Q/wt^ mice developed skin tumors (Fig. 2B) with an average of 7.6 and 11.7 palpable tumors/mouse (Fig. 2B), respectively. In contrast, at the same 17 week time point, the K14CreER^tam^;LSL-Nrf2^E79Q/wt^ mice developed an average of only 2.4 palpable tumors/mouse with an 82% tumor incidence (Fig. 2B), representing 70% inhibition in tumor promotion/development. From 19 to 39 weeks of TPA treatment, we also determined tumor incidence and multiplicity of tumors ≥1 mm diameter (Fig. 2C). Tumor incidence and multiplicity for the K14CreER^tam^;LSL-Nrf2^E79Q/wt^ mice remained decreased compared to the other groups for all palpable and for tumors ≥ 1 mm for the duration of the experiment (Fig. 2B–C). Tumor diameters were measured with digital calipers at 39 weeks of TPA treatment and mean skin tumor diameters for the K14CreER^tam^;LSL-Nrf2^E79Q/wt^ mice were significantly decreased (5 fold) compared to the tumors of K14CreER^tam^ and LSL-Nrf2^E79Q/wt^ mice (Fig. 2D). These results demonstrate skin tumor development/promotion is significantly inhibited by Nrf2^E79Q^ when expressed starting at 1 week after DMBA initiation.

### 3.3 Mutant Nrf2^E79Q^ expressing papillomas show increased progression to SCCs

**Fig. 3:**
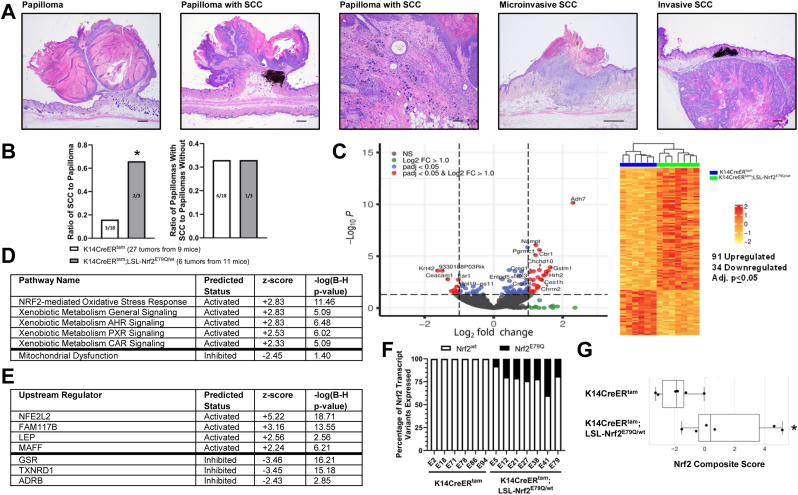
Tumors with *Nrf2*^*E79Q*^ activated post-DMBA display increased tumor progression and enriched *Nrf2* target gene expression. **(A)** Representative examples of an H&E-stained papilloma, papilloma with an area of SCC, micro-invasive SCC and invasive SCC. The scale bars are equal to 500um (papilloma, papilloma with SCC, microinvasive SCC and invasive SCC) and 250um (100× original magnification of papilloma with SCC) **(B)** Ratio of SCC to papilloma and ratio of papilloma with SCC to papilloma analyzed from tumors collected from 9 K14CreER^tam^ and 11 K14CreER^tam^;LSL-Nrf2^E79Q/wt^ mice. * Denotes p = 0.13 Fisher's Exact Test and p = 0.08 Risk Difference. **(C)** Volcano plot and heatmap of tumor RNAseq data from K14CreER^tam^;LSL-Nrf2^E79Q/wt^ skin tumors compared to K14CreER^tam^ skin tumors. Each column represents a tumor from a different mouse. **(D)** IPA pathway analysis of RNAseq data reveals, of the significantly enriched pathways, the top pathways predicted to be activated (z-score ≥2.0 are predicted to be activated) and pathways predicted to be inhibited (z-score ≤2.0) in K14CreER^tam^;LSL-Nrf2^E79Q/wt^ skin tumors compared to K14CreER^tam^ skin tumors. Right tailed Fisher Exact Test with Benjamini-Hochberg (B–H) multiple hypothesis testing-corrected p-value **(E)** IPA's upstream regulator analysis of RNAseq data reveals top upstream transcription regulators, upstream regulators with z-scores ≥2.0 are predicted to be activated and upstream regulators with z-scores ≤2.0 are predicted to be inhibited based on observed gene expression changes in tumors of K14CreER^tam^;LSL-Nrf2^E79Q/wt^ mice compared to K14CreER^tam^ mice. Right tailed Fisher Exact Test with Benjamini-Hochberg (B–H) multiple hypothesis testing-corrected p-value **(F)** Tumors from K14CreER^tam^;LSL-Nrf2^E79Q/wt^ epidermis expresses *Nrf2*^*E79Q*^ transcripts. Each bar represents a tumor from a different mouse. **(G)** OIS-PRM targeted proteomics showed a significant increase in the Nrf2 score in tumors from K14CreER^tam^;LSL-Nrf2^E79Q/wt^ mice compared to tumors from K14CreER^tam^ mice. * denotes p ≤ 0.05 via Mann-Whitney *U* test.

As described above, the expression of mutant Nrf2^E79Q^ in mouse epidermis after DMBA initiation inhibited skin tumor development (Fig. 2). However, we did observe skin tumors in K14CreER^tam^;LSL-Nrf2^E79Q/wt^ at the termination of the tumor experiment (40 weeks of TPA treatment). To identify potential differences in the tumors from K14CreER^tam^ and K14CreER^tam^;LSL-Nrf2^E79Q/wt^, we carried out histopathological analysis, RNAseq, and OIS-PRM targeted proteomics on representative tissue samples. Skin lesions were scored lesions as papilloma, papilloma with an area of SCC, micro-invasive SCC or an invasive SCC (Fig. 3A). NRF2^E79Q^ enhanced the progression/development of cutaneous SCC (Fig. 3B). K14CreER^tam^;LSL-Nrf2^E79Q/wt^ mice displayed a 4-fold increase in the ratio of SCCs/papillomas compared to K14CreER^tam^ mice. No differences between these groups were observed in the ratio of papillomas with an area of SCC/papillomas (Fig. 3B). Thus, activation of NRF2^E79Q^ before tumor promotion enhanced the progression to SCCs.

We next looked for differences in gene and protein expression between tumors of KK14CreER^tam^ mice vs. tumors from K14CreER^tam^;LSL-NRF2^E79Q/wt^ mice. RNAseq analysis identified 125 unique genes (91 upregulated and 34 downregulated) out of a data set of 14,002 genes that were altered in the tumors from K14CreER^tam^;LSL-Nrf2^E79Q/wt^ mice compared to the tumors from K14CreER^tam^ mice (adj. p ≤ 0.05) (Fig. 3C–Supplementary Table 5). IPA Pathway Analysis demonstrated an enrichment of NRF2 target genes in skin tumors of the K14CreER^tam^;LSL-Nrf2^E79Q/wt^ mice with the NRF2-mediated oxidative stress response canonical pathway found as the most enriched pathway (Supplementary Table 6). Of the significantly enriched pathways, the top pathways predicted to be activated (z-score ≥2.0) were pathways associated with NRF2 activation and the pathway predicted to be inhibited is mitochondrial dysfunction (Fig. 3D, Supplementary Table 6). IPA's Upstream Regulator Analysis identified NFE2L2 (NRF2) as the top upstream transcription regulator with the highest positive activation z-score and GSR with highest inhibition z-score that explained our dataset's observed gene expression changes (Fig. 3E–Supplementary Table 7). IGV analysis of *Nrf2* transcripts demonstrated that the mutant *Nrf2* transgene transcripts were expressed in all tumors of the TMX-treated K14CreER^tam^;LSL-Nrf2^E79Q/wt^ mice, with an average of 22% Nrf2 transcripts per tumor being the mutant (Fig. 3F). The overabundance of wild-type transcript is likely due to the contribution of stromal and vascular components of the tumor. To confirm and extend the RNAseq profiles to protein, we used a recently reported NRF2 centric OIS-PRM targeted proteomics assay [42]. As expected, we observed a statistically significant (p ≤ 0.05) increase in NRF2 protein levels and an Nrf2 activity signature score in in K14CreER^tam^;LSL-Nrf2^E79Q/wt^ mice, the latter of which is a composite score of the protein expression levels of canonical NRF2 target genes (Fig. 3G–Supplementary Table 8).

### 3.4 Activation of mutant Nrf2^E79Q^ causes tumor regression in pre-existing skin tumors

**Fig. 4:**
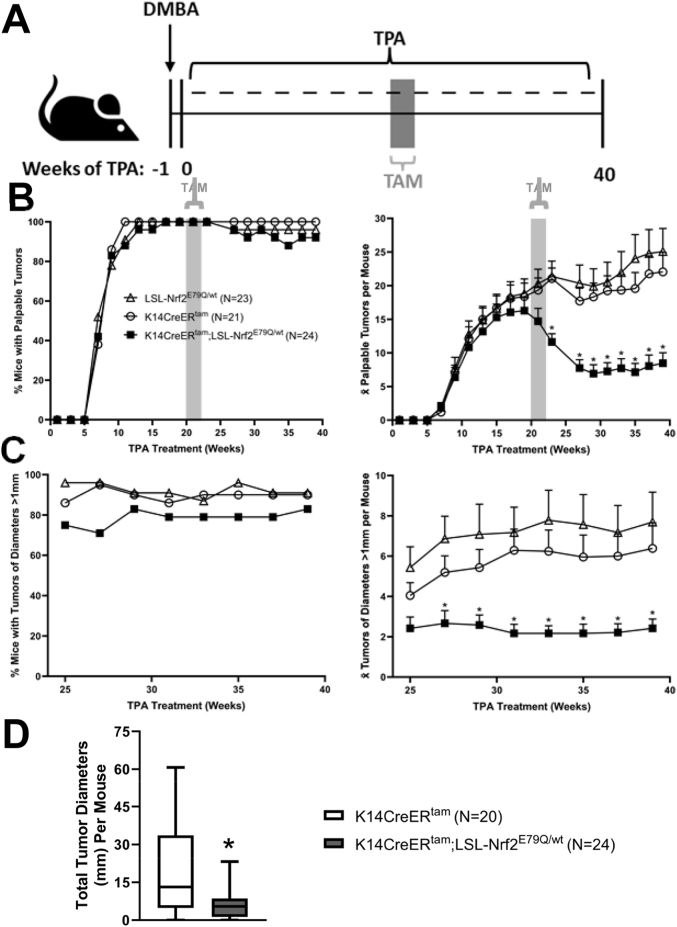
*Nrf2*^*E79Q*^ activation in pre-existing tumors causes tumor regression. **(A)** K14CreER^tam^, K14CreER^tam^;LSL-Nrf2^E79Q/wt^ and LSL-Nrf2^E79Q/wt^ mice were initiated with a single topical application of 200 nmol DMBA. One week later the mice were promoted with 10 nmol TPA thrice weekly for 40 weeks. After 20 weeks of TPA promotion the mice were dosed with 2.5 mg TMX i.p. once a day, 5 days a week for two weeks while continuing TPA promotion. Tumors were measured and counted every two weeks during TPA promotion. After 40 weeks of promotion tumors and whole epidermis were collected for histological, protein, and RNA analysis. **(B)** The incidence and average number of palpable skin tumors per mouse according to genotype. K14CreER^tam^;LSL-Nrf2^E79Q/wt^ mice had significantly less tumors than K14CreER^tam^ mice. LSL-Nrf2^E79Q/wt^ mice did not have significantly more tumors than K14CreER^tam^. *Denotes p ≤ 0.05 Student's t-test. **(C)** The incidence and average number of skin tumors >1 mm^3^ per mouse according to genotype. K14CreER^tam^;LSL-Nrf2^E79Q/wt^ mice had significantly less tumors than K14CreER^tam^ mice. LSL-Nrf2^E79Q/wt^ mice did not have significantly more tumors than K14CreER^tam^. *Denotes p ≤ 0.05 Student's t-test. **(D)** Diameters of skin tumors >1 mm^3^ per mouse were measured and grouped according to genotype. K14CreER^tam^;LSL-Nrf2^E79Q/wt^ mice had significantly less sum tumor diameter than K14CreER^tam^ mice *Denotes p ≤ 0.05 Student's t-test.

After determining the effect NRF2^E79Q^ on tumor promotion/development, we next examined the effect of NRF2^E79Q^ on preexisting DMBA-initiated/TPA promoted skin papilloma. After 20 weeks of TPA treatment when tumor incidence was 100% in all groups, TMX was administered to activate Nrf2^E79Q^ expression(Fig. 4A–B). TPA treatment was continued through to 40 weeks. By 7 weeks after the cessation of TMX treatment 60% of the tumors had regressed in K14CreER^tam^;LSL-Nrf2^E79Q/wt^ mice (Fig. 4B). We did not observe any tumor regression after TMX treatment in either the K14CreER^tam^ mice or the LSL-Nrf2^E79Q/wt^ mice. Instead, tumor multiplicity increased in both these groups of mice throughout the experiment (Fig. 4B). During 25–39 weeks of TPA treatment, we also determined tumor incidence and multiplicity of tumors ≥1 mm diameter (Fig. 4C). Tumor incidence and multiplicity in K14CreER^tam^;LSL-Nrf2^E79Q/wt^ mice remained lower than K14CreER^tam^ and LSL-Nrf2^E79Q/wt^ mice for all palpable tumors and for tumors ≥ 1 mm in diameter for the remainder of the experiment (Fig. 4B–C). At 39 weeks of TPA treatment, mean skin tumor diameters were significantly decreased (3 fold) in the K14CreER^tam^;LSL-Nrf2^E79Q/wt^ compared to the tumors of K14CreER^tam^ mice (Fig. 4D). These results demonstrate that activation NRF2^E79Q^ expression in pre-existing skin tumors rapidly inhibited their progression, leading to tumor regression in over half of the lesions.

### 3.5 Skin tumors resistant to tumor regression induced by Nrf2^E70Q^ expression display increased progression to SCCs

**Fig. 5:**
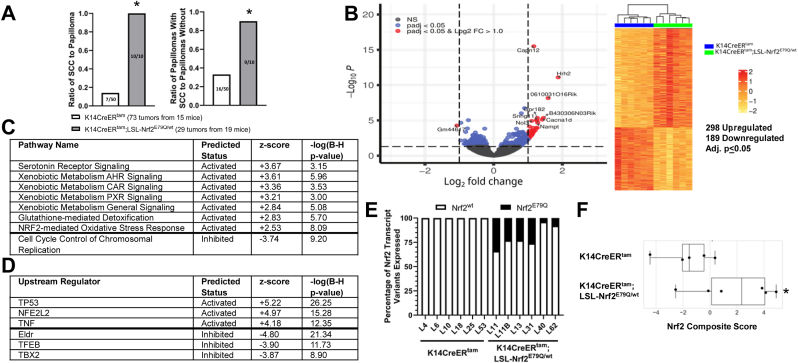
Remaining *Nrf2*^*E79Q*^ activated tumors display enhanced progression and enrichment of *Nrf2* target gene expression. **(A**) Ratio of SCC to papilloma and ratio of papilloma with SCC to papilloma from tumors collected from 15 K14CreER^tam^ and 19 K14CreER^tam^;LSL-Nrf2^E79Q/wt^ mice as determined by H&E histological analysis. * Denotes p ≤ 0.05 Fisher's Exact Test and p ≤ 0.05 Risk Difference. **(B)** Volcano plot and heatmap of tumor RNAseq data from K14CreER^tam^;LSL-Nrf2^E79Q/wt^ skin tumors compared to K14CreER^tam^ skin tumors. Each column represents a tumor from a different mouse. **(C)** IPA pathway analysis of RNAseq data reveals, of the significantly enriched pathways, the top pathways predicted to be activated (z-score ≥2.0) and pathways predicted to be inhibited (z-score ≤2.0) in K14CreER^tam^;LSL-Nrf2^E79Q/wt^ skin tumors compared to K14CreER^tam^ skin tumors. Right tailed Fisher Exact Test with Benjamini-Hochberg (B–H) multiple hypothesis testing-corrected p-value **(D)** IPA's upstream regulator analysis of RNAseq data reveals top upstream transcription regulators, upstream regulators with z-scores ≥2.0 are predicted to be activated and upstream regulators with z-scores ≤2.0 are predicted to be inhibited based on observed gene expression changes in tumors of K14CreER^tam^;LSL-Nrf2^E79Q/wt^ mice compared to K14CreER^tam^ mice. Right tailed Fisher Exact Test with Benjamini-Hochberg (B–H) multiple hypothesis testing-corrected p-value **(E)** Tumors from K14CreER^tam^;LSL-Nrf2^E79Q/wt^ epidermis expresses *Nrf2*^*E79Q*^ transcripts. Each bar represents a tumor from a different mouse. **(F)** OIS-PRM targeted proteomics showed an increase in the Nrf2 score in tumors from K14CreER^tam^;LSL-Nrf2^E79Q/wt^ mice compared to tumors from K14CreER^tam^ mice. * Denotes p = 0.08 via Mann-Whitney *U* test.

While 60% of the tumors regressed in K14CreER^tam^;LSL-Nrf2^E79Q/wt^ mice, 40% did not (Fig. 4B). We again characterized skin tumors from K14CreER^tam^ and K14CreER^tam^;LSL-Nrf2^E79Q/wt^ mice at the termination of the experiment (40 weeks of TPA treatment) by histopathological analyses, RNAseq and OIS-PRM targeted proteomics. Compared to K14CreER^tam^ mice, K14CreER^tam^;LSL-Nrf2^E79Q/wt^ mice displayed a 7-fold increase in the ratio of SCCs/papillomas and a 3-fold increase in the ratio of papillomas with an area of SCC/papillomas (Fig. 5A). RNAseq analysis identified 487 unique genes (298 upregulated and 189 downregulated) out of a data set of 13,140 genes that were altered in the tumors from K14CreER^tam^;LSL-Nrf2^E79Q/wt^ mice compared to the tumors from K14CreER^tam^ mice (adj. p ≤ 0.05) (Fig. 5B–Supplementary Table 9), a larger number of changes compared to the early TMX treatment groups (Fig. 3B). IPA Pathway Analysis again demonstrated an enrichment of NRF2 target genes in the tumors of the K14CreER^tam^;LSL-Nrf2^E79Q/wt^ mice with the kinetochore metaphase signaling pathway as the most enriched and the NRF2-mediated oxidative stress response canonical pathway as the third most enriched pathway (Supplementary Table 10). Of the significantly enriched pathways, the top pathways predicted to be activated (z-score ≥2.0) were serotonin receptor signaling and pathways associated with NRF2 activation. The pathways predicted to be most inhibited (z-score ≤ -2.0) included cell cycle control of chromosomal replication. (Fig. 5C–Supplementary Table 10). IPA's Upstream Regulator Analysis identified TP53 as the top upstream transcriptional regulator with NFE2L2 (NRF2) having a slightly lower Z-score (Fig. 5D–Supplementary Table 11). IGV analysis of Nrf2 transcripts demonstrated that the mutant Nrf2 transgene transcripts were expressed in all tumors of the TMX-treated K14CreER^tam^;LSL-Nrf2^E79Q/wt^ mice, with an average of 18% of the transcripts coming from the mutant allele (Fig. 5E). OIS-PRM targeted proteomics showed an increase the Nrf2 composite score that trended towards significance (p = 0.08) (Fig. 5F–Supplementary Table 8).

### 3.6 Mutant NRF2^E79Q^ activation early after DMBA increases the frequency of non-canonical ras mutations

**Fig. 6:**
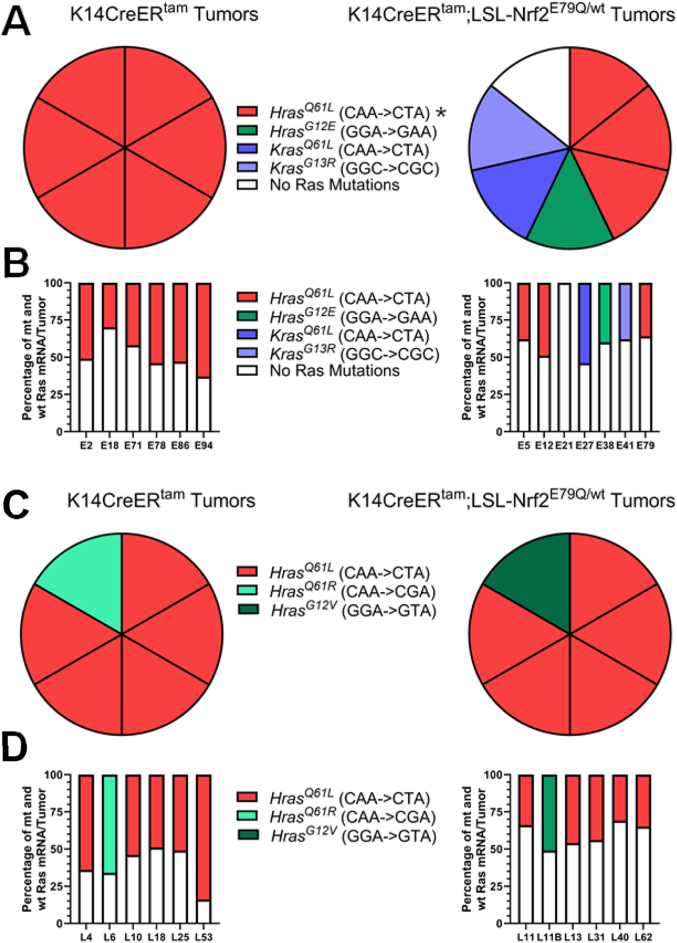
*Nrf2*^*E79Q*^ activation early after DMBA increases the frequency of non-signature H and K*Ras* mutations in tumors. **(A)** Presence of mutant *Ras* transcripts in 6 K14CreER^tam^ and 7 K14CreER^tam^;LSL-Nrf2^E79Q/wt^ skin tumors of early TMX treated mice collected for RNAseq. *Hras*^Q61L^ mutations were significantly altered in the K14CreER^tam^;LSL-Nrf2^E79Q/wt^ mice. *Denotes p ≤ 0.05 Fisher's Exact Test. **(B)** Percentage of the specific mutated *Ras* isoform mRNA and it's wild type isoform mRNA for each tumor in Fig. 6A. **(C)** Presence of mutant *Ras* transcripts in 6 K14CreER^tam^ and 6 K14CreER^tam^;LSL-Nrf2^E79Q/wt^ skin tumors of late TNX treated mice collected for RNAseq. **(D)** Percentage of the specific mutated *Ras* isoform mRNA and it's wild type isoform mRNA for each tumor in Fig. 6C.

Previous reports have established that DMBA produces the canonical oncogenic mutation A->T (Q61L) transversion in the 61st codon of *Hras* (*Hras*^*Q61L*^) that serves as the driver mutation in 95–100% of DMBA/TPA tumors [[25], [26], [27], [28], [29], [30]]. In agreement with these previous studies, IGV analyses of K, H and Nras mRNAs showed expression of the expected canonical *Hras*^*Q61L*^ mutation in all six (100%) of early TMX-K14CreER^tam^ tumors analyzed (Fig. 6A). Surprisingly, only 3/7 (43%) of K14CreER^tam^;Nrf2^LSL−E79Q/wt^ tumors contained the canonical *Hras*^*Q61L*^ mutations analyses; the remaining 57% displayed noncanonical *Hras* and *Kras* mutations or no *Ras* mutations (Fig. 6A). One tumor displayed a *Hras*^*G12E*^ mutations, one tumor displayed a *Kras*^*G13R*^ mutation, one tumor displayed a *Kras*^*Q61L*^ mutation and one tumor displayed no *H, K* or *Nras* mutations (Fig. 6A). The percentage for the specific mutated *Ras* mRNA isoform and its wild type isoform mRNA for each tumor in Fig. 6A is shown in Fig. 6B. No *Trp53* mutations were observed in any tumors and *Trp53* transcript levels were similar in all tumors. Our results indicate expression of mutant NRF2^E79Q^ starting one week after DMBA initiation inhibits the development of 70% of tumors. However, the tumors that do develop display an altered frequency and type of Ras mutations as well as an enhanced tumor progression to cutaneous SCC. In contrast, activation of the mutant Nrf2^E79Q^ allele in preexisting tumors did not significantly alter the Ras mutation signatures. As shown in Figs. 6B and 5/6 of the K14CreER^tam^ tumors analyzed contained the expected *Hras*^*Q61L*^ mutation while the other tumor possessed the closely related *Hras*^*Q61R*^ mutation. Similarly, 5/6 of the K14CreER^tam^;LSL-Nrf2^E79Q/wt^ tumors contained the expected *Hras*^*Q61L*^ mutation with the other tumor displaying a *Hras*^*G12V*^ mutation (Fig. 5B). The percentage for the specific mutated *Ras* mRNA isoform and it's wild type isoform mRNA for each tumor in Fig. 6C is shown in Fig. 6D. No *Trp53* mutations were observed in any tumors and *Trp53* transcript levels were similar in all tumors. Thus, the expression of mutant NRF2^E79Q^ in preexisting skin tumors causes regression of 60% of the tumors, however, the tumors that do develop display the expected canonical *Hras*^*Q61L*^ mutation at the expected frequency.

## 4 Discussion

The functional consequences of activating mutations in Nrf2, such Nrf2^E79Q^, on specific stages of tumor development is poorly understood. Using the stochastic mouse skin multistage carcinogenesis model with a DMBA-initiation/TPA-promotion protocol we characterized the effect of Nrf2^E79Q^ expression on distinct stages of tumor development. Among the advantages of this model is that tumor development and tumor regression can be visually monitored real time over long periods of time on the same mouse. To examine the effect of Nrf2^E79Q^ during promotion, Nrf2^E79Q^ expression was temporally and conditionally activated in the epidermis after DMBA initiation but before cutaneous tumor development. Nrf2^E79Q^ expression in epidermal keratinocytes was a potent inhibitor of tumor promotion, inhibiting promotion of 70% of skin tumors. The skin tumors that did develop showed enriched expression of Nrf2 target genes, expression of the mutant Nrf2^E79Q^ transcript and enhanced progression to SCCs. Surprisingly, only 43% of tumors analyzed contained the canonical signature *Hras*^*Q61L*^ mutation, with the remaining 57% displaying non-canonical *HRas* and *Kras* mutations or no *Ras* mutations. These results were unexpected as the frequency of *Hras*^*Q61L*^ mutation in skin tumors in the control K14CreER^tam^ group was 100% (Fig. 6A) matching historical data of skin tumors resulting from the DMBA-initiation/TPA-promotion in mice [[25], [26], [27], [28], [29], [30]].

*RAS* genes are mutated in ∼20% of human cancers [43]. The three RAS genes, *KRAS, NRAS* and *HRAS,* each have three hotspot mutation positions: codon 12, codon 13 and codon 61 [44]. Genomic analyses of many cancer types revealed multi-level specificity for; 1) the presence of any *RAS* mutation, 2) a preference for mutations in specific RAS genes and 3) biased prevalence for specific codon mutations [44,45]. For example, certain cancers almost always contain a mutant *RAS* like pancreatic cancer, while other cancers rarely contain a mutated *RAS*, such as breast cancer [45]. Further, *KRAS* is mutated in pancreatic and lung cancer, *NRAS* in melanoma and *HRAS* in oral and skin SCC. Finally, pancreatic cancer favors 12th codon mutations while lung favors 61st codon mutation [44,45]. Our result demonstrate mutant Nrf2^E79Q^ governs the Ras isoform, the codon and the substitution position bias in skin tumors when Nrf2 is activated early after initiation. Importantly, these bias changes result from mutant Nrf2^E79Q^ expressed from the endogenous *Nrf2* promoter allowing for physiological expression in an intrinsic keratinocyte manner early after initiation. The timing of when mutant Nrf2 is expressed in tumor development is critical for the *Ras* bias changes. When *Nrf2*^*E79Q*^ expression is turned on in pre-existing DMBA-initiated/TPA-promoted skin tumors, NRF2^E79Q^ does not affect the *Ras* isoform, position, and substitution bias. Nevertheless, when mutant Nrf2 is turned on early in skin after DMBA or late in pre-existing tumors the tumors that remain display similar progression to SCC suggesting that differences in Ras mutation and isoform profile may not further influence progression of skin papilloma to SCCs.

Deep sequencing of adult human normal tissues has revealed a mutational landscape composed of hundreds to thousands of somatic mutations in tissues such as skin [46], esophagus [47], colon [48], and lung [49]. Some of these mutations appear in cancer driver genes, and yet these tissues remain phenotypically “normal”. These findings are consistent with the notion that “initiated” human cells and tissues can maintain a normal phenotype until an endogenous or exogenous promoting stimulus provides favorable conditions for their expansion and transition to become a tumor [31]. Similarly, in the mouse skin model, the mutational landscape in DMBA-initiated mouse skin showed thousands of DMBA-induced mutations including the *Hras*^*Q61L*^ mutation [31]. Yet, the skin remains “normal” until tumor promotion begins. In our early NRF2^E79Q^ temporal expression model, the mutant *Nrf2*^*E79Q*^ was not expressed until well after the DMBA-induced mutagenesis. Therefore, the observed effects of NRF2^E79Q^ on inhibition of tumor promotion, enhanced progression and the altered *Ras* bias are independent of an effect of NRF2^E79Q^ on initiation but dependent on the pre-existing DMBA-mutational landscape and tumor promotion. Our finding suggests that the expression of Nrf2^E79Q^ early during promotion may create a favorable environment for the expansion of keratinocytes with non-canonical mutations *in Kras* and *Hras*. According to the *Ras* sweet-spot model [45], if RAS signaling and/or levels are too low, tumors will not develop, and if Ras expression/signaling are too high, senescence and apoptosis can occur blocking tumor development. The sweet spot represents the optimal conditions for Ras tumorigenesis, and NRF2^E79Q^ may modify the sweet spot or optimal conditions to favor tumor develop by different Ras isoforms and mutations. While these conditions are poorly understood, they may account for the preference of *KRAS* mutations in the pancreas, colon and lung cancer but not in skin and oral carcinomas. This tissue specificity in patients mirrors cancer mouse models. Additionally, activating mutations in KEAP1/NRF2 pathway often co-exist with *KRAS* mutations [50] and recently NRF2 activity levels have been shown to change the Kras mutation pattern at codon 61 in urethane-induced mouse lung carcinogenesis [51]. Understanding how Nrf2^E79Q^ can promote the expansion of mutant *Kras* keratinocytes in the mouse skin multistage model instead of the canonical *Hras*^*Q61L*^ mutation could shed light on mechanism driving tissue bias for *Kras* mutations. Such an understanding could reveal potential targets for therapy that could interfere with *Kras* tumorigenesis.

When mutant Nrf2 expression was activated in pre-existing DMBA-initiated/TPA-promoted tumors it caused regression of 60% of the tumors. The skin tumors that did not regress showed enrichment of expression of Nrf2 target genes, expression of the mutant Nrf2^E79Q^ transcript and enhanced progression to SCCs. Recent studies comparing lung adenocarcinoma development in the Kras^G12D/+^;p53^fl/fl^ GEMM with either *Keap1*^*R554Q*^ or *Nrf2*^*D29H*^ expressed under the control of the endogenous Nrf2 promoter found that the levels of Nrf2 pathway activity are important for tumor initiation, progression and histological grade [23]. With moderate NRF2 activation (e.g. heterozygous Nrf2^D29H^) initiation and early progression to low grade lung tumors was enhanced. At high levels of NRF2 activity tumor progression to higher histological grades was inhibited. Another recent study showed the co-expression of mutant Nrf2^L30F^ with Trp53^R172H^ caused esophageal SCC-like lesions while wildtype NRF2 hyperactivation induced by the loss of KEAP1 in the presence of TRP53^R172H^ does not, suggesting a gain of function for the mutant Nrf2^L30F^ [52]. Similarly, in our study, it is possible that different levels of NRF2 signaling have different effects on tumor development. For example, certain NRF2 signaling levels may result in inhibition of skin tumor promotion and cause tumor regression while others NRF2 signaling levels may enhance progression. This is somewhat analogous to the sweet spot model for Ras described above. Consistent with this notion, we observed an ∼50% decrease in mutant Nrf2^E79Q^ transcripts in papillomas/SCC compared to epidermis suggesting that this level of expression could be more conducive to tumor development and progression. On the other hand, this decrease could be due to the heterogeneity of the tumors as they contain stroma/vascular, immune cells etc. which express wildtype Nrf2 which would result in a dilution of mutant Nrf2 transcript. It is also possible some tumors cells may lose mutant Nrf2 expression, and this could be important in progression. Future studies using single cell sequencing of tumors could resolve these possibilities. Activated NR2 signaling can modify tumorigenesis through diminution of cellular tumor stress, altering the redox state of the cell, promoting immune evasion and metabolic reprograming [18,19,53,54]. Future studies will be aimed at understanding how mutant NRF2 alters the Ras isoform, position, and substitution bias in tumors and how it modifies the distinct stages of tumorigenesis.

## CRediT authorship contribution statement

**John G. Witherspoon:** Writing – review & editing, Writing – original draft, Visualization, Validation, Methodology, Investigation, Formal analysis. **Jonathan R. Hall:** Writing – review & editing, Investigation, Formal analysis. **Dereje Jima:** Visualization, Formal analysis. **Hannah M. Atkins:** Writing – review & editing, Methodology, Investigation. **Nathan T. Wamsley:** Writing – review & editing, Investigation. **Michael B. Major:** Writing – review & editing, Visualization, Methodology, Investigation. **Bernard E. Weissman:** Writing – review & editing, Resources, Project administration, Methodology, Funding acquisition, Conceptualization. **Robert C. Smart:** Writing – review & editing, Writing – original draft, Visualization, Validation, Supervision, Resources, Project administration, Methodology, Investigation, Funding acquisition, Conceptualization.

## Declaration of competing interest

All authors have declared they have no competing interests.

## Data Availability

No data was used for the research described in the article.
