# Supplementary material for: Mutant Nrf2E79Q enhances the promotion and progression of a subset of oncogenic Ras keratinocytes and skin tumors
Source: Redox Biol. 2024 Jun 28;75:103261. doi: 10.1016/j.redox.2024.103261 (PMC11269801; doi:10.1016/j.redox.2024.103261)
Supplement: Multimedia component 1 [file mmc1.docx]

Supplemental Tables

Table 1 RNAseq Mutant Nrf2 vs K14Cre

Table 2 RNAseq CDDO vs K14Cre

Table 3 Pathway Analysis IPA Mutant Nrf2 vs Cre

Table 4 Upstream Regulators IPA Mutand Nrf2 vs Cre

Table 5 RNAseq Early TMX - Mutant vs K14Cre tumors

Table 6 Pathway Analysis IPA Early TMX - Mutant vs K14Cre tumors

Table 7 Upstream Regulators IPA Early TMX - Mutant vs K14Cre tumors

Table 8 Protein Level Abundances

Table 9 RNAseq Late TMX - Mutant vs K14Cre tumors

Table 10 Pathway Analysis IPA Late TMX - Mutant vs K14Cre tumors

Table 11 Upstream Regulators IPA Late TMX - Mutant vs K14Cre tumors
